# Strategic Combination of DNA-Damaging Agent and PARP Inhibitor Results in Enhanced Cytotoxicity

**DOI:** 10.3389/fonc.2013.00257

**Published:** 2013-09-30

**Authors:** Julie K. Horton, Samuel H. Wilson

**Affiliations:** ^1^Laboratory of Structural Biology, NIEHS, National Institutes of Health, Research Triangle Park, NC, USA

**Keywords:** DNA polymerase β, XRCC1, PARP-1, PARP inhibitors, base excision repair

## Abstract

PARP inhibitors (PARPi) are under clinical trial for combination cancer chemotherapy. In the presence of a PARPi, PARP-1 binds DNA strand breaks but cannot produce poly(ADP-ribose) polymers or undergo auto-poly(ADP-ribosyl)ation. DNA binding is persistent, hindering DNA repair. Methylated bases formed as a result of cellular exposure to DNA-methylating agents are repaired by DNA polymerase β (pol β)-dependent base excision repair (BER) producing a 5′-deoxyribose phosphate (5′-dRP) repair intermediate. PARP-1 binds and is activated by the 5′-dRP, and PARPi-mediated sensitization to methylating agents is considerable, especially in pol β-deficient cells. Cells deficient in the BER factor XRCC1 are less sensitized by PARPi than are wild-type cells. PARPi sensitization is reduced in cells expressing forms of XRCC1 deficient in interaction with either pol β or PARP-1. In contrast, agents producing oxidative DNA damage and 3′- rather than 5′-repair intermediates are modestly PARPi sensitized. We summarize PARPi experiments in mouse fibroblasts and confirm the importance of the 5′-dRP repair intermediate and functional pol β and XRCC1 proteins. Understanding the chemistry of repair is key to enhancing the clinical success of PARPi.

## Background

Clinical trials suggest that PARP inhibitors (PARPi) may represent an opportunity to gain selective killing of cancer cells, since the cytotoxic effects make use of deficiencies in cellular DNA repair systems that are distinctive for individual tumor cells versus normal tissues (1, 2). But it has proved difficult to design chemotherapy regimes because of toxic side effects such as myelosuppression. Information enabling prediction of PARPi effects is not easy to gain from the literature and may not be well recognized in the community. We suggest that understanding PARPi effects in model systems, such as mouse embryonic fibroblast (MEF) cells in culture, will be informative for considering strategies in cancer chemotherapy. We have discussed this viewpoint in a recent article (3). Here, we summarize current experiments with the aim of understanding the roles of PARP in mammalian cell DNA repair and how the presence of the inhibited PARP-1 protein during base excision repair (BER) may promote cell killing. The level of cell killing observed with DNA-damaging agents is modulated by co-treatment with a PARPi and by expression of other BER proteins such as XRCC1 and pol β, and we will outline a model to explain these effects. Selection of specific chemotherapeutic agents combined with specific repair deficiencies in patients may prove to be extremely beneficial.

## BER of Base Damage and Binding of PARP-1 to Intermediates of BER

The mammalian BER pathway is important for the removal of single base lesions in double-stranded genomic DNA. Base damage can arise through spontaneous base loss from DNA or from base alkylation and oxidation from both endogenous and exogenous sources. Methyl methanesulfonate (MMS) is a directly acting DNA-methylating agent causing alkylation of base nitrogens (e.g., 7-methylguanine), whereas the oxidizing agent peroxynitrite produces reactive oxygen species (ROS) that oxidize DNA bases resulting in the promutagenic DNA lesion 8-oxoguanine and other base lesions. During single-nucleotide BER of a methylated base, repair is initiated by a lesion-specific monofunctional glycosylase (i.e., *N*-methylpurine DNA glycosylase; MPG), that removes the damaged base leaving an abasic (AP) site in double-stranded DNA. The DNA backbone is then incised 5′ of the AP site by AP endonuclease 1 (APE1) resulting in a 1-nucleotide (nt) gap with margins of 3′-OH and 5′-deoxyribose phosphate (dRP) groups. DNA polymerase β (pol β) binds to this repair intermediate, removes the 5′-dRP group and performs single-nucleotide gap filling DNA synthesis. Many of the glycosylases specific for oxidative DNA damage (e.g., 8-oxoguanine DNA glycosylase; OGG1) are bifunctional enzymes that have an associated AP lyase activity in addition to their glycosylase activity. After base removal, this activity cleaves the DNA backbone 3′ to the abasic site leaving 3′-dRP and 5′-PO_4_ margins in a single-nucleotide gap. APE1 is able to remove the 3′-blocking group leaving a 3′-OH-containing substrate suitable for DNA synthesis and ligation. In this BER sub-pathway there will be no formation of a 5′-deoxyribose-containing blocking group or requirement for pol β-dependent dRP lyase tailoring activity to enable DNA ligation (4).

PARP-1 is an abundant nuclear protein involved in DNA damage recognition. It can bind to AP sites and single-strand breaks in DNA, including the 5′-dRP-containing intermediate of BER of MMS-induced damage. Once bound to DNA, PARP-1 becomes catalytically activated synthesizing poly(ADP-ribose) (PAR) polymers from NAD^+^, and resulting in poly(ADP-ribosyl)ation of itself, as well as other proteins involved in DNA repair and chromatin remodeling (5, 6). PARP-1, the first discovered member of a family of proteins, is responsible for the majority of cellular PARP activity after DNA damage. Following auto-modification, PARP-1 can interact with other BER proteins such as XRCC1 and pol β enabling their recruitment to the damage site (7, 8). A recent publication has suggested that PARP-1 recruits XRCC1 to single-strand break repair, but not to sites of oxidative damage BER (9). This may be due the absence of 5′-dRP intermediate formation during oxidative damage (8-oxoguanine) repair (4).

In the case of methylation damage, after removal of the abasic site sugar by pol β lyase activity and completion of repair by pol β gap filling and DNA ligation, PARP-1 dissociates from DNA, and the PAR glycosidic bonds are rapidly cleaved, primarily by poly(ADP-ribose) glycohydrolase (PARG) (10). In earlier photoaffinity labeling studies, PARP-1 was identified as the predominant BER intermediate-binding factor in the MEF cell extract (11). Use of other binding ligands revealed PARP-1 binding specificity for the 5′-dRP-containing BER intermediate with much less binding when an alternate BER intermediate without the 5′-dRP group was used (12). The results are consistent with a biological role for an interaction between PARP-1 and the 5′-dRP-containing BER intermediate. Additionally, as discussed below and elsewhere (3), and in agreement with the *in vitro* studies, we find that the cytotoxic effects of cellular PARP inhibition correlate very well with the presence of the 5′-dRP group in the BER intermediate.

## PARP Inhibition and Hypersensitivity to DNA Damage

In the presence of a catalytic inhibitor, PARP-1 can still bind to DNA damage sites, but auto-ribosylation is prevented (1). In its inhibited and inactivated state, PARP-1 binding to DNA is stabilized, hindering the BER process (13). We have proposed that the DNA-bound and inhibited PARP-1 molecule results in cytotoxicity due to formation of replication-dependent double-strand breaks (DSBs) (14).

Experiments in MMS-treated MEFs demonstrated that PAR synthesis was completely inhibited by the PARPi 4-amino-1,8-naphthalimide (4-AN) (15, 16). Wild-type (WT) MEFs are highly (40-fold) sensitized to MMS and to the methylating chemotherapeutic agent temozolomide (TMZ) by 4-AN co-treatment (17). Positive TMZ/PARPi potentiation data have been reported in a number of other systems, e.g., human tumor cell lines and xenografts (18, 19), and this combination has been successful in phase I clinical trials in patients with solid tumors (20) or melanoma (21). Additionally, a recently reported phase II study of an inhibitory dose of a PARPi with TMZ in metastatic melanoma provided evidence for chemopotentiation and increased disease-free survival (22). The authors suggest the need for a phase III trial comparing TMZ with TMZ + PARPi, also for evaluation of DNA repair capacity in patients to identify those most likely to benefit from this combination.

In contrast to the results with TMZ and MMS, co-treatment with 4-AN has minimal effect (1.1-fold sensitization) on cellular sensitivity to the reactive oxidant peroxynitrite (17). This agent results in oxidative DNA modifications including 8-oxoguanine, 8-nitroguanine and single-strand breaks (23). Repair of 8-oxoguanine initiated by the bifunctional OGG1 is not expected to produce the 5′-dRP blocked repair intermediate. Thus, a key difference in BER following treatment with these two agents (MMS and peroxynitrite) is initiation by a monofunctional versus a bifunctional glycosylase. Only in the former case (repair of MMS damage by a monofunctional glycosylase) will there be formation of a repair intermediate with a 5′-sugar phosphate blocking group. The results emphasize that the presence of the 5′-dRP blocking group is critical for binding PARP-1 and for observing PARPi-mediated sensitization to DNA damage.

## PARP Inhibitor Effects in BER Protein-Deficient and Defective Cells

The most notable phenotype of pol β null MEFs is hypersensitivity to S*_N_*2 alkylating agents such as MMS, and to S*_N_*1 alkylating agents such as the chemotherapeutic methylating agent TMZ (24, 25). Hypersensitivity to these agents in pol β-deficient mouse fibroblasts can be reversed by expression of either the full-length protein or the 8 kDa dRP lyase domain with 5′-dRP gap-tailoring activity (26). XRCC1-deficient cells are extremely hypersensitive to monofunctional methylating agents including MMS and TMZ (4). XRCC1 interacts with a number of repair proteins and binding to PARP-1 is critical for recruitment of XRCC1 to damaged sites in DNA. Thus, in PARP-1-deficient cells, recruitment of XRCC1 is hindered (7). The interaction between the amino-terminal domain (NTD) of XRCC1 and the polymerase domain of pol β is essential for recruitment of pol β to sites of damaged DNA (27). Hypersensitivity to MMS can be reversed by transfection of full-length WT XRCC1 protein into *Xrcc1*^−/−^ cells (28), but as observed previously in CHO cells (29), only partial reversal is observed following expression of a mutant protein (V88R) that does not interact with pol β. Likewise, there is no rescue of hypersensitivity following expression of the L360R mutant XRCC1 protein that has disrupted folding of the BRCT I domain and interrupted interaction with PARP-1 (30, 31). The results suggest that interactions between PARP-1, XRCC1, and pol β are required for the protective effects of XRCC1 and pol β against MMS and TMZ exposures.

**Figure 1:**
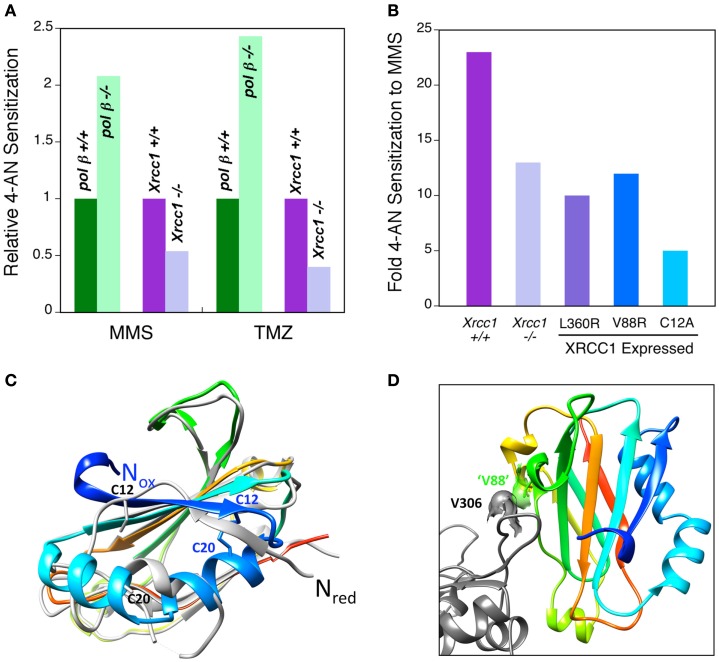
**PARPi-mediated sensitization to MMS and TMZ and ability of XRCC1 to interact with pol β**. **(A)** Relative sensitization in wild-type and repair protein-deficient MEFs (as indicated) by a 24 h exposure to the PARPi 4-AN. Pol β-deficient cells are more highly sensitized than the wild-type line (green), while XRCC1-deficient cells are less sensitized (purple). **(B)** Level of PARPi-mediated sensitization to MMS in *Xrcc1*^+^*^/^*^+^ (WT) and *Xrcc1*^−/−^(null) MEFs, and in XRCC1 null cells expressing mutated XRCC1 proteins (L360R, V88R and C12A) as indicated. **(C)** The XRCC1 NTD has been crystallized in two forms: oxidized and reduced (34). An overlay of the oxidized (colored, PDB ID 3LQC) and reduced (light gray, PDB ID 3K75) forms indicates that the amino-termini are on opposite sides of this domain (N_ox_ and N_red_, respectively). Accordingly, the interactions around the amino-termini are very different for these two forms. The cysteine residues (C12 and C20, respectively) that participate in disulfide bond formation in the oxidized form are indicated. **(D)** ‘V88’ (green) of mouse NTD forms a hydrophobic interaction with V306 (gray) of pol β. This portion of the pol β-binding interface is similar for both the oxidized and reduced forms of the NTD, and includes the hydrophobic interaction of XRCC1 ‘V88’ with V306 of pol β. V88 corresponds to V86 of the structurally characterized human NTD of XRCC1. Replacing this valine with arginine (V88R) significantly reduces the interaction between these proteins (28).

A high level of sensitization to MMS and TMZ is observed in both *pol β*^+^*^/^*^+^ and *pol β*^−/−^ MEFs following combination treatment with 4-AN. Interestingly, the level of sensitization of *pol β*^−/−^ cells is at least double that observed in *pol β*^+^*^/^*^+^ cells (Figure 1A). Thus, when utilizing the TMZ + PARPi combination, pol β null cells become considerably more TMZ-sensitive than WT cells. Similar pol β-dependent results were obtained with other agents (MMS, MNU) that result in DNA damage repaired by monofunctional glycosylase-initiated BER. We propose that through its role in removing the 5′-dRP intermediate, pol β is able to regulate the PARPi-mediated sensitization in TMZ cytotoxicity. There have been numerous reports of cancer related pol β single-nucleotide polymorphisms (32, 33). Expression of a dRP lyase inactivating mutation would be a critical biomarker for enhancement of TMZ + PARPi cytotoxicity. Additionally, current assays for dRP repair intermediates are used with cell culture models in laboratory research, but have not yet been adapted for clinical use. Such adaptation of these techniques represents an opportunity for translational research. Ongoing studies will address this question.

In contrast *Xrcc1*^+^*^/^*^+^ WT cells are more highly sensitized (two to threefold) to MMS and TMZ than are *Xrcc1*^−/−^cells (Figures 1A,B). Thus, the interaction between XRCC1 and PARP-1 proteins appears to be required for the strongest PARP-inhibitor-mediated sensitization. Expression of WT XRCC1 will stabilize the protein complex through its accessory protein functions, and this will allow for more efficient PARP binding to the 5′-dRP-containing BER intermediate. Another possibility, that XRCC1 may modulate the dRP lyase activity of pol β, is being tested in the laboratory. Sensitization in cells expressing the L360R mutated XRCC1 protein without interaction with PARP-1 (30, 31) was similar to that in *Xrcc1*^−/−^ cells (Figure 1B), consistent with the proposal that the interaction between XRCC1 and PARP-1 enables the sensitization. In *Xrcc1*^−/−^ cells expressing an XRCC1 mutant (V88R) that is compromised in its ability to bind pol β, sensitization to MMS was also about half of the level observed in WT cells (Figure 1B).

Pol β and XRCC1 interact through a redox-sensitive binding interface in the N-terminal domain (NTD) of XRCC1 (34), and equal levels of both oxidized and reduced forms of the full-length protein are found in untreated WT MEFs (28). Structural characterization of both oxidized and reduced forms of the XRCC1 NTD reveal that they have distinct conformations (Figure 1C) and a different pol β functional interaction, with the oxidized form binding tighter to pol β (34). The disulfide bond between C12 and C20 required for stabilizing the oxidized form is evident in the structure shown, whereas C12 and C20 are far apart in the reduced form (Figure 1C). Nevertheless, some portions of the pol β-binding interface are similar for both the oxidized and reduced forms of the NTD, and this includes the hydrophobic interaction between V306 (Figure 1D) of pol β and V88 of mouse XRCC1 NTD (“V88”). Cells expressing C12A XRCC1 protein locked in the reduced state are equally as MMS resistant as WT cells (28). However, cells expressing reduced C12A XRCC1 have a considerably lower level of PARPi-mediated sensitization than WT cells (5- and 23-fold, respectively) (Figure 1B). These results are consistent with the requirement for tight XRCC1-pol β interaction for strong PARPi-mediated sensitization (V88R in Figure 1B). However, the extremely low PARPi-mediated sensitization in the cells expressing the reduced XRCC1 protein suggests there may be additional XRCC1 effects linked to its ability to take the oxidized form.

Pol β null cells are minimally hypersensitive to DNA oxidants such as peroxynitrite, IR, and bleomycin where repair of oxidative DNA damage does not involve significant formation of an intermediate with a 5′-sugar phosphate. The low PARPi sensitization observed in WT cells for peroxynitrite co-treatment was also seen in pol β-deficient cells (17), and similar data (≤3-fold sensitization) were obtained for clinically utilized IR and the radiomimetic agent bleomycin. Bleomycin results in formation of ROS, oxidized sugars and abasic sites with 3′-blocking groups such as 3′-phosphoglycolate (35), and repair may involve pol β and BER, but the 5′-sugar phosphate blocking group is not abundantly formed. Again the results suggest a requirement for a 5′-sugar phosphate-containing repair intermediate for significant cellular hypersensitivity in pol β-deficient cells. Similarly, despite the hypersensitivity of *Xrcc1*^−/−^ cells to methylating agents, only low-level hypersensitivity is observed to oxidative DNA damage (4).

Taken together, these results are consistent with a correlation between formation of the 5′-dRP blocking group and the degree of PARPi-mediated sensitization. In the absence of pol β, cells will be deficient in the 5′-dRP gap-tailoring activity, allowing for enhanced binding of PARP-1 to DNA damage and for more PARPi-mediated sensitization. These cells therefore demonstrate the concept of synthetic lethality occurring under conditions of PARP inhibition in the presence of pol β-deficiency. The notion of synthetic lethality explains the vulnerability of cells that are deficient in one pathway in repair (here pol β-mediated BER) and then have repair additionally blocked by a chemical agent (e.g., a PARPi). A similar well-appreciated situation occurs when PARPi are used in BRCA- and other homologous recombination-deficient cells and tumors (36–38). The expression level of specific repair proteins is expected to modulate the degree of PARPi-mediated sensitization. The chemistry of DNA damage and repair also regulates PARPi effects, since in the absence of the 5′-dRP group-containing repair intermediate, there is minimal PARPi-mediated sensitization.

## Model for PARP Inhibitor-Mediated Cell Killing

PARP inhibitors have become valuable in chemotherapy as part of a combination regime or as monotherapy. In MEF model systems, the magnitude of the cell killing effect of a PARPi in combination with a genotoxic agent is dependent on the chemistry of the DNA repair intermediate. Inhibition of PARP when it is bound to a 5′-dRP group-containing intermediate results in a dramatic cell sensitization. In contrast, if the repair intermediate does not have the 5′-dRP group, both PARP-1 binding and inhibitor-mediated sensitization are minimal.

**Figure 2:**
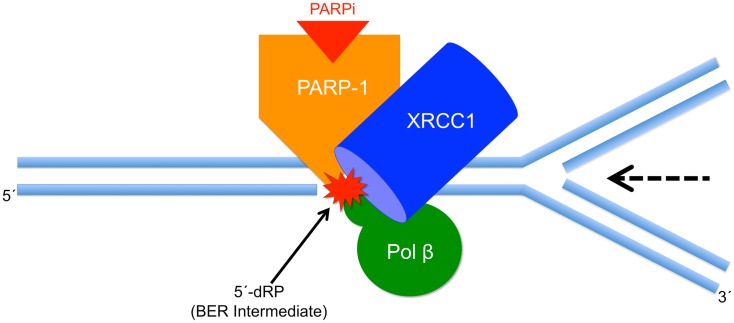
**Schematic model illustrating PARPi-mediated cell killing**. Shown is a replication fork colliding with the BER repair complex bound at the 5′-dRP of the BER intermediate. The replication fork moves in the direction indicated by the arrow and becomes stalled at the protein complex. We propose that stalling leads to replication fork collapse, DSB formation, and cell death.

A schematic model consistent with these results is shown in Figure 2. It is important to note that the current results do not prove this model, but instead the model is useful as a framework for designing future experiments. The model illustrates a replication fork colliding with the BER repair protein complex bound at the 5′-dRP-containing site in double-stranded genomic DNA. The replication fork moves in the direction of the arrow and becomes stalled at the protein complex, consisting of PARP-1, pol β, and XRCC1, among other proteins not shown in the image. Replication fork stalling is proposed to lead to fork collapse, DSB formation, and eventually to cell death. Thus, fork stalling is proportional to cell killing, at least in the context of this model. The model predicts that in the absence of inhibited PARP-1 or the 5′-dRP group, the protein complex will not form.

Pol β is able to remove the 5′-dRP group from repair intermediates. In pol β null BER-deficient MEFs, excess 5′-dRP group-containing intermediates may accumulate, and PARP-1 binding and PARPi-mediated sensitization will be considerable. The model illustrates that the dRP group is key for PARP-1 binding, such that in the absence of pol β dRP lyase activity, there is more PARP-1 binding and more PARPi-induced cell killing. In the absence of XRCC1, pol β binding at damaged DNA is decreased and this is expected to lead to diminished dRP group removal and more cell killing. Further, the model predicts that in the absence of XRCC1 the stability of the complex will be reduced, and consequently the replication fork may be able to bypass the complex without stalling. The weaker affinity of the reduced form of XRCC1 for pol β is consistent with a less stable overall complex, more replication fork bypass, and less PARPi-mediated cell killing as observed experimentally. The results are consistent with this prediction in that the absence of XRCC1 expression, or less binding of XRCC1 to PARP-1 or pol β, is associated with lower PARPi-mediated sensitization.

In summary, PARPi are under study for use in cancer chemotherapy and here we report that the ability for PARPi-induced sensitization in model mammalian cell lines (mouse fibroblasts) correlates with the chemistry of DNA repair intermediates. Surprisingly, we find that in the absence of the 5′-dRP group-containing repair intermediate, there is minimal PARPi-mediated sensitization. Additionally, we show that the presence of functional BER factors pol β and XRCC1 regulate PARPi-induced sensitization, but this is only under conditions where the 5′-dRP group is formed.

## Conflict of Interest Statement

The authors declare that the research was conducted in the absence of any commercial or financial relationships that could be construed as a potential conflict of interest.
